# Partial trisomy of 1q42.1 and 8q24.3 deletion: a family history

**DOI:** 10.1590/1984-0462/2026/44/2025354

**Published:** 2026-08-24

**Authors:** Natasha Malgarezi de Moraes, Marcela Rodrigues Nunes, Rafaella Mergener, Ana Kalise Böttcher da Silveira, Helena Froener Peruzzo, Lívia Polisseni Cotta Nascimento, Mariluce Riegel, Paulo Ricardo Gazzola Zen

**Affiliations:** a Universidade Federal de Ciências da Saúde de Porto Alegre, Porto Alegre, RS, Brazil.; b Instituto Nacional de Ciência e Tecnologia em Multiômica Aplicada à Saúde de Precisão, Porto Alegre, RS, Brazil.

**Keywords:** Partial trisomy, Case report, Chromosomal translocation, Maternal inheritance, Trissomia parcial, Relato de caso, Translocação cromossômica, Herança materna

## Abstract

**Objective:**

To evaluate the effects of an unbalanced inheritance in a patient, while investigating and elucidating family history.

**Case description:**

This is a case report of a patient carrying an unbalanced combination consisting of a partial trisomy of 1q42.1 and a microdeletion in 8q24.3, resulting from the segregation of the familial balanced reciprocal translocation between chromosomes 1 and 8.

**Comments:**

A genetic investigation was carried out in the family, elucidating the segregation mechanisms, possible outcomes, and inheritance lineage of this balanced translocation and its unbalanced forms. This case report was the first involving these two specific chromosomal regions.

## RESUMO

**Objetivo:**

Investigar as consequências da translocação desbalanceada em uma paciente, enquanto se investiga e elucida o histórico familiar do caso.

**Descrição do caso:**

Trata-se do caso de uma paciente com uma combinação desbalanceada da trissomia parcial da região 1q42.1 e microdeleção da região 8q24.3, resultante da segregação de uma translocação recíproca balanceada familiar entre os cromossomos 1 e 8.

**Comentários:**

Uma investigação genética foi realizada na família, elucidando os mecanismos de segregação, os possíveis resultados e a linhagem hereditária dessa translocação balanceada e suas formas desbalanceadas. Este relato de caso foi o primeiro envolvendo essas duas regiões cromossômicas específicas.

## INTRODUCTION

Recombination between homologous chromosomes is normal, occurs during meiosis, and grants genetic diversity and transmission of new combinations of linked alleles to the next generation.^1^ However, recombination between non-allelic regions can result in chromosomal alterations. Chromosomal alterations can occur *de novo*, when observed for the first time in a family, or can be inherited. *De novo* rearrangements can be recurrent, with similar breakpoints, sizes, and genomic content shared by unrelated individuals, or non-recurrent with unique parameters.^2^ They can present variable clinical features, expressivity, modifier genes, or genetic content, such as reciprocal translocations, a balanced rearrangement that can lead to a unbalanced offspring.

Chromosomal translocation occurs when a chromosomal region breaks, generating two fragments, and either of these fragments reattaches to a new chromosome. This alteration has a higher potential for heritability. In this case, the offspring can inherit the same parental translocation, a variant form thereof or an unbalanced combination. Carriers of chromosomal translocation have reproductive difficulties, phenotypic abnormalities, and higher risks of miscarriages.^3^ Within the human genome, some regions are more susceptible to breaks and rearrangements when exposed to specific deoxyribonucleic acid (DNA)-binding or DNA replication-inhibiting compounds; these regions are called common fragile sites.^4^ Common fragile sites, spread across the genome, present common characteristics such as high flexibility, fragility, low stability, and delayed replication, making them more susceptible to breaks and rearrangements.^5^

A case report is presented of a patient carrying an unbalanced inherited translocation resulting in a partial trisomy of chromosome 1q42.1 and, consequently, a microdeletion in chromosome 8q24.3. Family history revealed a balanced reciprocal translocation between chromosomes 1 and 8, associated with cases of miscarriages and unbalanced offspring across generations.

## CASE REPORT

The female patient was born at 36 weeks of gestation by urgent cesarean section delivery. Regarding obstetric history, the patient is the result of the mother’s second pregnancy, the first being a spontaneous abortion. Subsequently, she was referred to the Genetics department because of her family history of chromosomal abnormality. Her mother presented a reciprocal chromosomal translocation between chromosomes 1 and 8 [46, XX,t(1;8)(q42.1;q24.3)] inherited from her grandmother; one aunt and a great-aunt also have this translocation. The daughter’s blood karyotype was performed and showed partial trisomy of 1q42.1 and, consequently, monosomy of 8q24.3 [46, XX, der(8)t(1;8)(q42.1;q24.3)dmat].

**Figure 1:**
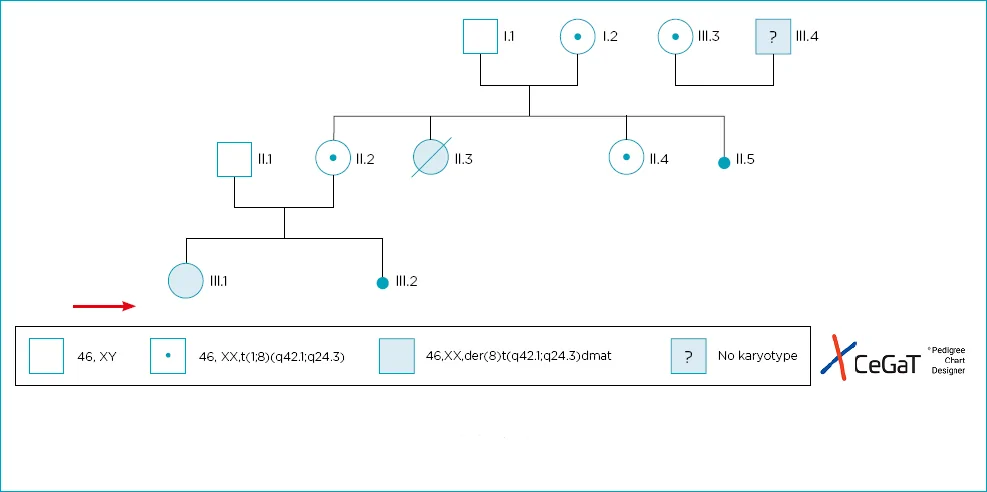
Family pedigree.

The mother had been a patient at the Clinical Genetics Service at Hospital Irmandade Santa Casa de Misericórdia de Porto Alegre (ISCMPA) and at the cytogenetics laboratory at Universidade Federal de Ciências da Saúde de Porto Alegre (UFCSPA) since 1994. She started a genetic investigation because her sister, who passed away during infancy, was born with multiple malformations, global developmental delay, and developed an abdominal malignant tumor. At that time, the patient’s maternal grandparents (I.1 and I.2) were referred for genetic evaluation, prompting a systematic family-wide cytogenetic investigation. Conventional karyotype analysis was performed in both grandparents and their two daughters (II.2, the proband’s mother, and II.3, a maternal aunt who died in infancy), following informed consent. After the identification of a familial chromosomal rearrangement, extended family members were subsequently evaluated. These results enabled the characterization of the segregation pattern and the construction of the pedigree presented in Figure 1, which illustrates the cytogenetic findings for each evaluated family member.

Subjects with a dot: 46, XX,t(1;8)(q42.1;q24.3); Subjects in color: 46,XX,der(8)t(1;8)(q42.1;q24.3)mat; Subjects with a question mark didn’t perform a karyotype analysis; Subjects without any marker or color: 46,XY; Red arrow: proband.

FISH: fluorescence *in situ* hybridization.

**Source:** Produced by the authors using CeGat software.

**Figure 2:**
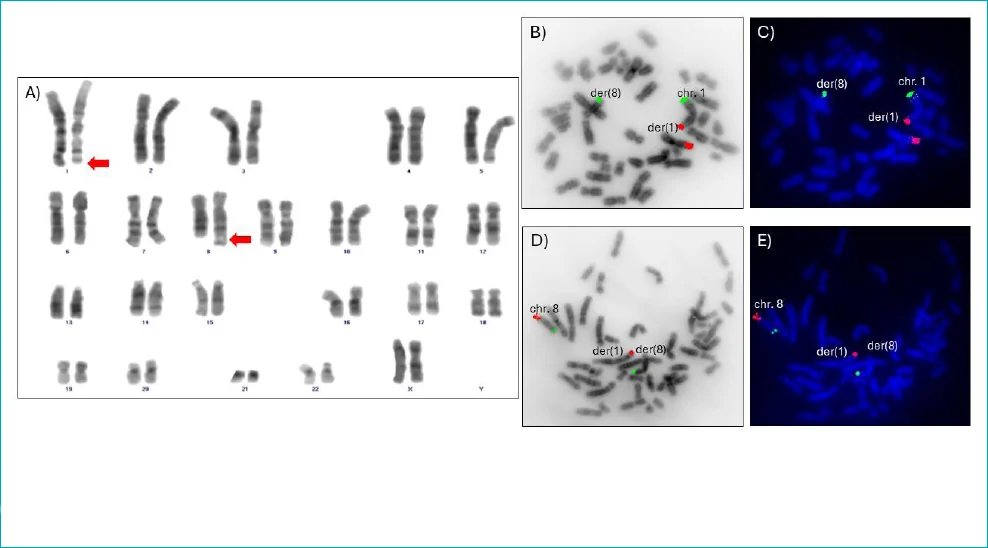
Fluorescence *in situ* hybridization analysis of the mother.

**Figure 3:**
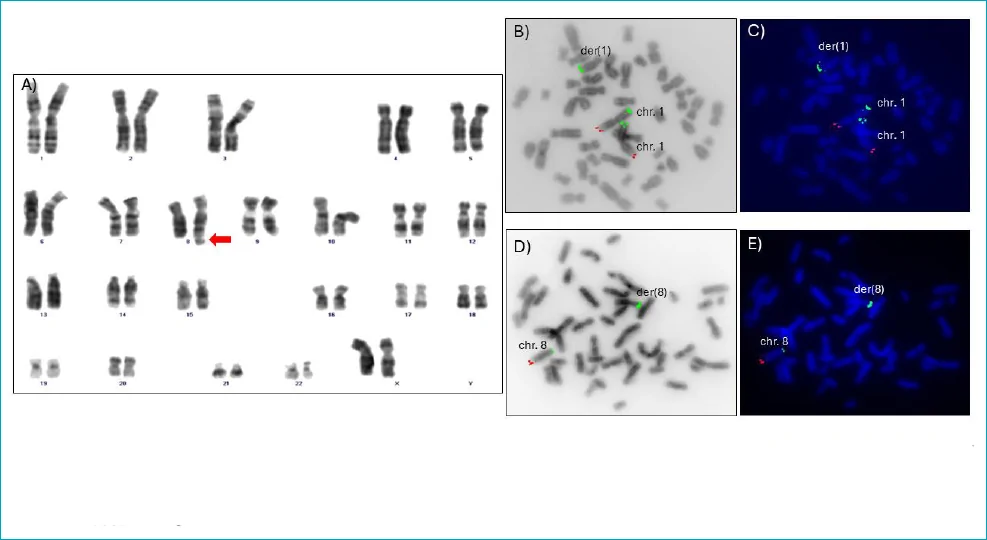
Fluorescence *in situ* hybridization analysis of the proband.

In 1994, the karyotype was performed at the UFCSPA laboratory. Recently, both karyotype and fluorescence *in situ* hybridization (FISH) analyses were performed on the proband and her mother in the Anthony Daher Laboratory at Casa dos Raros in partnership with the UFCSPA laboratory. All techniques followed internal protocols. Peripheral blood lymphocytes were cultured for 72 hours in RPMI-1640 medium supplemented with fetal bovine serum and stimulated with phytohemagglutinin. Chromosomes were harvested following standard colchicine arrest, hypotonic treatment (KCl 0.075M), and methanol/acetic acid fixation. GTG-banding (Giemsa-Trypsin-Giemsa) was performed, and karyotypes were analyzed at a resolution of approximately 400–550 bands, according to the International System for Human Cytogenomic Nomenclature (ISCN) 2024.^6^ The FISH analysis was performed using three different probes: the Monosomy 1p36 Deletion FISH Probe (Cytocell, Oxford Gene Technology, Cambridge, UK) for the 1p36.33 region (red signal) and the 1q44 region (green signal); the 8pter probe (Cytocell, Oxford Gene Technology, Cambridge, UK) for the p-arm subtelomere region (green) and the 8qter probe (Vysis, Abbott Molecular, Illinois, USA) for the q-arm subtelomere region (orange), to confirm the regions of the translocation, partial trisomy, and microdeletion in the proband (Figure 2) and her mother (Figure 3).

A) Karyotype of the mother with the result 46,XX,t(1;8)(q42.1;q24.3)—red arrows indicate the translocated regions; B and C) FISH analysis with the probes 1p36.33 region (red) and the 1q44 region (green) colored with black/white and DAPI filters, respectively; D and E) FISH analysis with the probes 8pter region (green) and the 8qter region (red) colored with black/white and DAPI filters, respectively.

FISH: fluorescence *in situ* hybridization.

**Source:** Produced by the authors.

A) Karyotype of the proband with the result 46,XX,der(8)t(1;8)(q42.1;q24.3)mat—the red arrow indicates the partial trisomy and microdeletion region; B and C) FISH analysis with the probes 1p36.33 region (red) and the 1q44 region (green) colored with black/white and DAPI filters, respectively; D and E) FISH analysis with the probes 8pter region (green) and the 8qter region (red) colored with black/white and DAPI filters, respectively.

FISH: fluorescence *in situ* hybridization.

**Source:** Produced by the authors.

The karyotype of the patient’s mother was repeated after the one performed in 1994 to improve the quality of the images and confirm the 46,XX,t(1;8)(q42.1;q24.3) results. The patient’s karyotype was also performed, showing 46,XX,der(8)t(1;8)(q42.1;q24.3)mat. The FISH analysis of the mother showed the reciprocal translocation between chromosomes 1q44 and 8qter. The patient’s FISH analysis showed a partial trisomy of the 1q44 region and a microdeletion of the 8qter region.

The patient, now 10 years old, has a global developmental delay, associated with autism spectrum disorder, intellectual deficit, and some dysmorphic features such as macrocephaly. She has a history of frequent upper respiratory tract infections and one episode of a tonic-clonic seizure when she was 1 year old. A brain magnetic resonance imaging performed at the time revealed thinning of the corpus callosum, ectasia of the supratentorial ventricles, and a hyperintense signal adjacent to the trigones of the lateral ventricles; the electroencephalogram was normal. During follow-up, significant changes in growth and dysmorphic aspects were observed. Physical examination revealed dysmorphic features such as low-set ears with unfolded helices, high palate, sacral pit, and bilateral equinovarus feet. Serial anthropometric data demonstrate a consistent growth pattern characterized by macrocephaly and increased stature. At 1 year and 8 months, the patient measured 83 cm in length and had a head circumference of 51 cm (>97th percentile). At 3 years and 6 months, height was 102 cm, weight 15.3 kg, and head circumference 53.5 cm (>98th percentile).

At 6 years of age, weight ranged from 20.25 to 24.3 kg and height from 115 to 122 cm, although measurements were obtained with some difficulty. At 7 years, she weighed 30 kg, measured 130 cm in height, and had a head circumference of 57 cm (>98th percentile). At 9 years of age, her recorded weight was 34 kg and head circumference was 55 cm (remaining >98th percentile). On follow-up, head circumference remained >98th percentile, confirming persistent macrocephaly. Height remained >95th percentile, while weight was between the 90th and 95th percentiles, indicating proportional overgrowth.

## DISCUSSION

Partial trisomy of chromosome 1q is a rare finding in patients; in the literature, only a few cases were reported with the same region as the present case, but all of them were translocated with different chromosomes or regions than the patient in this case report and her family.^7-9^ Curatolo et al. studied the molecular composition of the DNA and multiple common fragile sites present in the human genome. Among the regions analyzed by the authors, three were located in 1q42.1 and one was positioned at the boundary between 1q42.1 and 1q42.2.^10^ This finding may have facilitated the break and rearrangement observed in the present family.

In 2009, Verheij et al. reported two patients with an interstitial microdeletion of 8.35 Mb in chromosome 8q24, resulting in a phenotype of coloboma, congenital heart disease, physical dysmorphia, psychomotor delay, and seizures.^11^ More cases with similar microdeletions have been described since then, and the most commonly deleted region is 78 kb and includes three critical genes: *SCRIB*, *NRBP2*, and *PUF60*; the haploinsufficiency of one or more of these genes is responsible for the phenotype.^12^ The proband shared the same clinical findings as all patients described with 1q42.1 partial trisomy, such as developmental delay, macrocephaly, and facial dysmorphia.^7-9^ Furthermore, the proband also shares some phenotypes present in patients with 8q24.3 deletions, like intellectual delay, seizures, autism, and recurrent infections.

The proband’s karyotype, 46,XX,der(8)t(1;8)(q42.1;q24.3)mat, reveals an unbalanced chromosomal rearrangement derived from the balanced reciprocal translocation identified in the mother, 46,XX,t(1;8)(q42.1;q24.3), which in turn was inherited from the maternal grandmother, who also carries the same balanced rearrangement. This three-generation pedigree demonstrates that the translocation t(1;8)(q42.1;q24.3) is a familial rearrangement transmitted through the maternal lineage.

The three-generation transmission observed in this family is particularly informative. The maternal grandmother transmitted the balanced translocation to the mother without clinical consequence, consistent with the well-established observation that balanced translocation carriers are phenotypically normal. However, when the mother — herself a carrier — underwent meiosis, she produced an unbalanced gamete carrying the der(8), which, upon fertilization, gave rise to the proband’s unbalanced karyotype. This pattern illustrates a key feature of familial reciprocal translocations: the same rearrangement can be stably transmitted as a balanced form across generations, while simultaneously carrying a significant risk of generating unbalanced offspring in each carrier pregnancy.^13,14^

During meiosis, in any balanced carrier of this translocation, including the mother and potentially other family members identified through testing, the two normal chromosomes (1 and 8) and the two derivative chromosomes [der(1) and der(8)] form a quadrivalent structure at meiosis I.^15^ This quadrivalent can segregate through several modes, each yielding gametes with distinct chromosomal constitutions. In total, up to 32 different meiotic outcomes are theoretically possible from a reciprocal translocation quadrivalent, of which only two result in a normal or balanced complement.^15^

The meiotic segregation of this translocation can yield multiple distinct chromosomal outcomes in every carrier pregnancy. Only alternate segregation products result in a chromosomally balanced and phenotypically normal individual. The proband’s unbalanced karyotype is consistent with adjacent-1 segregation, which is the most clinically significant source of liveborn unbalanced offspring in reciprocal translocation families.^16,17^ In adjacent-1 segregation (unbalanced outcomes), the homologous centromeres segregate to opposite poles, but non-homologous chromosomes co-segregate. This produces two types of unbalanced gametes: one carrying der(8) without der(1), resulting in partial trisomy 1q (q42.1→qter) and partial monosomy 8q (q24.3→qter)—the imbalance observed in the proband—and one carrying der(1) without der(8), resulting in partial monosomy 1q and partial trisomy 8q. Adjacent-1 segregation is the most common source of unbalanced offspring among reciprocal translocation carriers, with reported frequencies ranging from approximately 22 to 38% of gametes.^16,17^

One family member carrying the unbalanced karyotype — a maternal aunt of the proband — reportedly developed an abdominal malignant tumor and died in infancy in 1994. The absence of comprehensive clinical records for this family member precludes definitive conclusions; nonetheless, the development of a malignant tumor in a carrier of the unbalanced translocation highlights the importance of considering the oncological relevance of 8q deletions. Deletions at 8q24.11–q24.13 have been associated with Langer-Giedion syndrome (LGS, OMIM #150230), a contiguous gene deletion syndrome caused by haploinsufficiency of *TRPS1* and *EXT1*, which predisposes affected individuals to multiple cartilaginous exostoses and carries a recognized risk of malignant transformation to chondrosarcoma or osteosarcoma.^18^ The deletion in the proband and her deceased aunt involves the more distal 8q24.3 region, which is distinct from the classical LGS critical region; however, the broader 8q24 locus is well established as a region of genomic instability and cancer susceptibility, with associations reported across multiple tumor types, including colorectal, prostate, breast, and ovarian cancers.^19,20^ Due to the absence of histopathological confirmation and the limited clinical data available, a definitive causal relationship between the chromosomal rearrangement and the reported family member’s neoplasia cannot be established. Nevertheless, oncological surveillance may be considered in the long-term follow-up of individuals carrying unbalanced forms of this translocation.

Female carriers of reciprocal translocations, such as the mother and grandmother in this family, have been reported to have a higher rate of transmitting unbalanced forms and a higher frequency of 3:1 and 4:0 segregation products compared to male carriers.^21,22^ This sex-specific difference in segregation behavior is particularly relevant for reproductive risk assessment in this family, where the translocation has been transmitted exclusively through the female line.

The genomic imbalances expected from the various segregation modes of this translocation provide a biological explanation for pregnancy loss. Given that chromosome 1 is the largest human autosome and is highly gene-dense throughout its length — including the distal 1q region — partial trisomy or monosomy of 1q segments is likely to be poorly tolerated, resulting in early embryonic lethality or spontaneous miscarriage. Similarly, partial imbalances of chromosome 8q, particularly involving the 8q24 region, which harbors several developmentally critical genes, may also contribute to pregnancy failure.^21^ The fact that the proband survived to term with the der(8) unbalanced karyotype suggests that this particular imbalance is among the more tolerable outcomes of this translocation, while other segregation products are likely to be uniformly lethal in utero.

In this family, the recurrence risk applies not only to future pregnancies of the mother (46,XX,t(1;8)(q42.1;q24.3), but also to any sibling of the mother who inherits the balanced translocation from the grandmother, who would also face equivalent reproductive risks and should be offered genetic testing and counseling. Genetic counseling is strongly recommended for all known and potential carriers within this family. Prenatal diagnosis through chorionic villus sampling or amniocentesis should be offered in all future pregnancies of carrier women, allowing karyotypic analysis of the fetus. Preimplantation genetic testing for structural rearrangements is a well-established reproductive alternative for translocation carriers, enabling the selection of balanced or normal embryos prior to implantation and substantially reducing both the risk of pregnancy loss and the birth of chromosomally abnormal children.^23-25^

This report describes a three-generation family harboring a familial reciprocal translocation t(1;8)(q42.1;q24.3), which resulted in an unbalanced karyotype — partial trisomy of 1q42.1 and deletion of 8q24.3 — in the proband. The chromosomal breakpoint in the 1q41–1q42 junction region likely corresponds to a known common fragile site, potentially facilitating this rearrangement. To our knowledge, this is the first reported case involving these two specific chromosomal regions. Recognition of this condition carries dual clinical importance: it allows for targeted, multidisciplinary developmental interventions to improve the quality of life of the affected child, and it enables comprehensive genetic counseling for the family, including reproductive risk assessment and access to prenatal or preimplantation genetic testing. In resource-limited settings where advanced molecular techniques such as microarrays or Optical Genome Mapping are unavailable, clinical cytogenetics combined with FISH remain a valuable diagnostic approach. This case reinforces the importance of karyotyping extended family members when a chromosomal rearrangement is identified, as carrier relatives face substantial reproductive risks that may benefit from appropriate genetic counseling and surveillance.

## Data Availability

The database that originated the article is available with the corresponding author.
